# Prenatal fructose exposure independently impacts placental phenotype and female offspring kidney function and liver composition in rats

**DOI:** 10.14814/phy2.70684

**Published:** 2025-11-30

**Authors:** A. Augusto Coppi, Clint Gray, Aliny A. B. Lobo Ladd, Fernando V. Lobo Ladd, Thamires Santos‐Silva, David S. Gardner

**Affiliations:** ^1^ Faculty and Health and Life Sciences University of Bristol Bristol UK; ^2^ School of Veterinary Medicine and Science University of Nottingham, Sutton Bonington Campus Sutton Bonington Leicestershire UK; ^3^ Gillies McIndoe Research Institute Wellington New Zealand; ^4^ Universidade Cidade de São Paulo São Paulo Brazil; ^5^ Department of Morphology/Laboratory of Neuroanatomy, Biosciences Center Federal University of Rio Grande Do Norte Natal Brazil; ^6^ Xenobr, São Paulo University São Paulo Brazil

**Keywords:** beverage, fructose, kidney, liver, rat

## Abstract

Archaeological and anthropological evidence suggests that human ancestors' diets were rich in fiber, potassium, and complex carbohydrates, while low in sodium, refined sugars, and energy density. Over time, agroindustrialization led to diets poorer in fiber and micronutrients but higher in sodium, simple sugars, and calorie‐dense foods. This shift contributed to the rise of noncommunicable diseases (NCDs) such as obesity, type 2 diabetes, and cardiovascular diseases, which now account for 70% of global premature deaths. Maternal nutrition affects fetal development and long‐term health. High sucrose or fructose intake during pregnancy can alter placental function, impacting fetal growth and metabolism. Placentae from male and female fetuses may respond differently to maternal diet. However, the effects of excessive maternal fructose intake on the placenta and offspring remain underexplored. In this study, rat dams consuming fructose‐sweetened beverages ate less food but drank more, significantly impacting placental volume and vascular structure. Long‐term effects on offspring were sex‐specific: females showed greater water retention and liver fat accumulation. High maternal fructose intake altered placental anatomy and had sex‐specific effects on kidney and liver function in adult offspring, even without further fructose exposure. These findings highlight the importance of maternal diet in preventing future metabolic diseases.

## 1 INTRODUCTION

Archaeological and anthropological studies suggest that the diet of human ancestors was rich in fiber, potassium, complex carbohydrates, and proteins, while relatively low in sodium, refined sugars, and energy density (Chrzan & Cargill, 2022; Konner & Eaton, 2023; Noakes et al., 2023). Analyses of modern hunter‐gatherer diets suggest that the plant‐to‐animal energy ratio in the paleolithic diet ranged from 5:1 to 1:1, with a predominant alkaline acid load, reflecting a high intake of fruits, vegetables, and lean proteins (Chrzan & Cargill, 2022; Lieberman et al., 2023; Pontzer & Wood, 2021; Singh & Singh, 2023).

Since this period, when physiological and metabolic systems were evolving, there has been a gradual shift away from the paleolithic diet. With the advent of agriculture approximately 10,000 years ago, and more intensively after the Industrial Revolution, the human diet became progressively lower in fiber and micronutrients while becoming richer in sodium, simple sugars, and high‐calorie density foods (Clemente‐Suárez et al., 2023; Zimmet et al., 2014). This mismatch between genetic evolution and changes in the modern food environment has been associated with an increasing prevalence of noncommunicable chronic diseases (NCDs), including obesity, type 2 diabetes, hypertension, and cardiovascular diseases (Clemente‐Suárez et al., 2023; Kuzawa & Manus, 2023; Mohajan & Mohajan, 2023).

Currently, NCDs are estimated to account for approximately 70% of all deaths worldwide, with substantial economic impacts (Lago‐Peñas et al., 2021; Luciani et al., 2023). In China, India, and the United Kingdom, the costs associated with NCDs reach US$ 558 billion, US$ 237 billion, and US$ 33 billion, respectively, while in the United States, annual expenditures on diabetes and hypertension exceed US$ 750 billion (Bloom et al., 2012; World Health Organization, 2005).

Diet modification is a cost‐effective strategy for preventing NCDs (Pereira et al., 2024; World Health Organization, 2005). Studies show that adopting a paleolithic diet, even in the short term, significantly reduces blood pressure, cholesterol, triglycerides, and insulin resistance (de la O et al., 2022; Koulouridis et al., 2023; Mubeen et al., 2023). Lowering salt intake to 3 g/day could prevent up to 92,000 deaths annually in the U.S. and generate savings of US$ 24 billion (Alonso et al., 2021; Bibbins‐Domingo et al., 2010). Moreover, reducing daily consumption of sugar‐sweetened beverages has been shown to lower systolic blood pressure (Chen et al., 2010; Hernández‐López et al., 2022).

Early intervention, such as a focus on improved nutrition during pregnancy, can also have lasting impacts, as maternal nutrition influences the risk of NCDs in the adult offspring—a concept known as the developmental programming of health and disease (Alabduljabbar et al., 2021; Reynolds et al., 2022). The placenta plays a key role in this context, being an intermediary between maternal diet and fetal growth and development. The placenta regulates the transfer of nutrients, hormones, and metabolites, directly influencing fetal growth and metabolic programming (de Araújo et al., 2023; Leonardi, 2022). Alterations in maternal diet, such as high consumption of simple sugars or salt, can modify placental function, affecting the availability of essential nutrients and predisposing the offspring to metabolic and cardiovascular disorders (Busnatu et al., 2022; Juul et al., 2021; Nutrition & Food Science, 1976). Furthermore, maternal diets rich in sucrose or fructose alone can disrupt the placental transport of glucose and amino acids, impacting fetal liver metabolism and contributing to intrauterine growth restriction (Bazer et al., 2024; Moses et al., 2022).

Another key factor is the placenta's role in modulating sexual dimorphisms in fetal development. Evidence suggests that male and female placentas respond differently to maternal nutritional challenges, potentially influencing susceptibility to metabolic and cardiovascular diseases later in life (Conlon & Arnold, 2023; Meakin et al., 2021; Munguia‐Galaviz et al., 2024). Therefore, consideration of sex‐specific responses to help clarify the mechanisms of fetal programming and their implications for male and female offspring health is important. To date, few studies have investigated the effects of maternal high intake of fructose on the placenta and on the longer‐term health of offspring in both sexes.

## 2 MATERIALS AND METHODS

### 2.1 Ethics

Animal procedures were carried out under license and in accordance with the Home Office Animals (Scientific Procedures) Act 1986, Project License [PPL‐40‐2925] and were approved by the local animal welfare and ethical review board of the University of Nottingham. At the end of all experiments, rats were euthanised in a sealed chamber using a rising concentration of CO_2_, followed by cervical dislocation after confirmation of circulatory arrest.

### 2.2 Experimental procedures‐dams

Sprague Dawley dams (190–200 g; 8–10 weeks of age) were kept in a temperature (20–22°C) and humidity (55%–65%) controlled environment and subjected to a 12‐hour light/dark cycle (0700–1900 h). Rats were randomly assigned to one of two treatment groups: (1) Control diet (CD; *n* = 12 dams), fed purified standard chow (TD.08164; Teklad Harlan, Maddison. WI.) and tap water; (2) Fructose diet (FD; *n* = 12 dams), fed purified standard chow (TD.08164) and tap water with 10% D‐fructose (Sigma‐Aldrich, UK) added. All rats were fed the experimental diets ad libitum for at least 28 days prior to conception and throughout gestation and lactation. At day 18 of gestation, a proportion of dams (*n* = 6/group) were acclimatized to a metabolic crate from which all urine was collected over a 24 h period (day 19 to day 20) to assess renal function. Afterward, these dams were euthanized for organ collection (maternal fat depots, liver, kidneys, and placentae) for assessment of fructose‐sweetened beverage effects on ectopic fat deposition (liver) and organ anatomy (placentae). Remaining dams (*n* = 6/group) continued on diet protocols to littering (standardized to 8 pups, 4 male/4 female per dam) through to weaning. At this time, offspring were housed according to sex and fed standard laboratory chow unless otherwise indicated.

### 2.3 Experimental procedures‐offspring

Between 8 and 14 weeks of age, two siblings from each litter (one male and one female) were entered into one of three protocols to assess: (1) baseline organ structure and composition – assessed after euthanasia without exposure to any novel diet or procedure (CD, male [*n* = 6] female [*n* = 5]; FD, male [*n* = 5] female [*n* = 5]); (2) baseline renal function—assessed by 24 h urine collection in a metabolic crate (after 24 h acclimatization to the environment) with a paired blood sample collected at 24 h (CD, male [*n* = 6] female [*n* = 5]; FD, male [*n* = 5] female [*n* = 5]); (3) response to fructose challenge—drinking water was substituted for fresh water with 10% fructose solution added for 5 days. On Day 4 of 5, the renal function was assessed as described above for dams.

### 2.4 Experimental measurements

All dams and/or offspring were euthanized between 09.00 and 11.00 h. Plasma or urinary osmolality was determined by freezing point depression (Osmomat 030, Gonotech, UK) with intra‐assay variability being <1%. Tissue dry weights were determined by freeze‐drying. Physiological measurements are presented adjusted to body weight (BW kg) to allow informed assessment of between sex differences (males being much larger than females). Urine flow rate (Vurine) was measured in mL per 24 h and is presented as mL/min/kg BW. Creatinine (Ccr), albumin (Calb), or osmolal (Cosm) clearance (mL/min/kg BW) were calculated as (e.g., for creatinine; ([Crurine* Vurine kg BW]/1440)/Crplasma). Free water clearance (CH_2_O) was calculated as urine flow rate – COsm.

### 2.5 Placental stereology

Three rat placentas (representing male and female fetuses) from each dam were sampled using a multistage systematic uniform random (SUR) design (Howard & Reed, 2018). Paraffin‐embedded placentas were fully serially sectioned at 5 μm, producing on average 600 sections per placenta. Sections were then collected onto glass slides and stained with Hematoxylin–Eosin to estimate the placental volume as well as the fractional total volumes of three placental compartments: maternal decidua, spongio‐trophoblast, and labyrinth. Images were acquired using a DM 6000 Leica microscope equipped with a High‐End DP 72 Olympus digital camera and projected onto a flat computer screen. Stereological analyses were performed using the new CAST Visiopharm stereology system version 4.4.4.0. For estimation of placenta volume (Vplacenta), the Cavalieri principle was applied with every 60th section sampled (Gundersen & ØSterby, 1981).

$$V\left(\text{placenta}\right)=T.\sum \text{Aplacenta}$$

where T is the between‐section distance (300 μm) and ∑Aplacenta is the sum of the delineated profile areas of placenta sections. Profile areas were estimated from the numbers of randomly positioned test points (~400 per placenta) hitting the whole reference space and a real equivalent of a test point. The mean volume shrinkage (coefficient of variation, CV, expressed as a decimal fraction of the mean) was estimated to be 23.8% (0.21) in CD and 13.2% (0.18) in FD. Correction for global shrinkage was applied prior to formal analysis of volume fractions. The fractional volumes of placenta occupied by its compartments: maternal decidua (Vv_MD_), spongio‐trophoblast (Vv_ST_), and labyrinth (Vv_L_) as well as the fractional volume of labyrinth blood vessels (Vv_LBV_) were estimated by point counting. Each grid of randomly positioned test points (~120 per placental compartment) was superimposed on the same sections used for the Cavalieri estimate. Points falling on each compartment, Pcompartment, and on the entire placenta (Pplacenta), were counted, and volume fractions (VV) of each compartment on a given placenta were estimated using the following equation:

$$\mathrm{Vv}≔\frac{\sum P\text{compartment}}{\sum P\text{placenta}}$$

Volume density varies from 0 to 1 and may be expressed as a percentage (Mayhew, 2008). The total volume of maternal decidua (V_MD_), spongio‐trophoblast (V_ST_), labyrinth (V_L_), and labyrinth vessels (V_LBV_) may be estimated by multiplying the respective fractional volume by the reference volume (Vplacenta) (Howard & Reed, 2018; Mayhew, 2008). The precision of a stereological estimate was expressed as a coefficient of error (CE) calculated as described by (Gundersen et al., 1999). When applying design‐based stereological methods, a reasonable precision of the stereological estimate is attained when CE is up to about half of the observed coefficient of variation (CVobs), (Campbell et al., 1970; Gundersen & ØSterby, 1981; Mathieu et al., 1983).

### 2.6 Analysis of liver composition

Dry mass was calculated by freeze drying a known mass of frozen liver tissue. Hepatic lipid content was assessed using a modification of Folch's method (Folch et al., 1957). In brief, 0.5–1.0 g hepatic tissue was homogenized in a total volume of 10 mL cold chloroform/methanol (2:1), agitated for 15 min at room temp followed by phase separation by centrifugation at 2400 rpm for 10 min. The concentration of triglycerides obtained from this reaction was determined by an automated colorimetric‐based assay (Rx‐IMOLA; SKU, TR3823, Randox Ltd., Country Antrim, UK) and the results were expressed as a concentration of milligrams of triglycerides per gram of tissue wet weight. The phospholipid composition of liver tissue was determined by gas chromatography in which any remaining chloroform was evaporated by applying a nitrogen stream and then 2 mL of hexane was added to each sample. The phospholipids were transmethylated by adding 40 μL of methyl acetate and 40 μL of methylation reagent (30% sodium methoxide, 4.1 mL methanol; Fisher Scientific Ltd. Loughborough, UK) to each reaction. Samples were left to react for 10 min at room temperature and the reaction was terminated through the addition of 60 μL of 0.2 g dried oxalic acid in 6 mL diethyl ether.

Methylated residues were extracted by adding 200 mg of calcium, then centrifugation (5 min at 2000 rpm). The fatty acid methyl esters were then injected (split ratio 50:1) into a gas chromatograph (GC 6890; Agilent Technologies Ltd., Stockport, UK). Separation of fatty acid methyl esters was performed with a Varian CP‐88 (Crawford Scientific™ Ltd., Strathaven, UK) capillary column with hydrogen as carrier gas. Oven temperature was programmed from 59°C to 100°C at 8°C per min, then to 170°C at 6°C per minute and held for 10 minutes. The temperature of the injector and detector was set at 255°C and 250°C. The fatty acid methyl esters were identified by comparing the retention times with a fatty acid methyl esters standard (Sigma‐Aldrich Co LLC, Gillingham, UK) and the area percentage in moles was used for the statistical analysis. A total of 37 fatty acids were extracted including: saturated fatty acids: C4:0, C6:0, C8:0, C10:0, C11:0, C12:0, C14:0, C15:0, C16:0, C17:0, C18:0, C20:0, C21:0, C22:0, C23:0, C24:0; monounsaturated fatty acids: C14:1, C15:1, C16:1, C17:1, C20:1, C24:1; polysaturated fatty acids, Omega (n)‐3: C18:3n3, C20:3n3, C20:5n3, C22:6n3 and Omega (n)‐6: C18:2n6t, C18:2n6, C20:3n6, C20:4n6. Omega (n)‐9: C18:1n9t, C18:1n9C, C20:1n9.

### 2.7 Liver histology

Paraformaldehyde (4% solution) fixed, and paraffin‐embedded sections were cut (5 μm) onto polysine slides (Menzel‐Glaser, GmbH & Co. Braunschweig, Germany) and stained with hematoxylin and eosin using standard histology protocols. Sections were visualized by light microscopy. Oil red O histochemical lipid staining was used to assess the intra‐hepatic lipid content. The stain detects fat droplets in cells, which stain with a red tint. In brief, 10 μm cryosections were placed in a Coplin jar containing 60% isopropanol for 15 min. Immediately afterwards, the sections were incubated for 20 min in a freshly prepared solution containing 0.5% w/v oil red‐O/isopropanol (Fisher Scientific Ltd. Loughborough, UK) diluted in a 1% w/v solution of dextrin. Thereafter, the stained samples were immersed for 1 min in 60% isopropanol and rinsed in cool running water. The sections were counterstained with Harris' hematoxylin for 3 min, followed by a short rinse in cool running water, and dipped in Scott's tap water (×3) and fast dried. Finally, a drop of 50% v/v glycerol‐water solution was placed over the samples to preserve the lipids, and the coverslip was sealed by applying an acetone‐based nail polish.

### 2.8 Statistics

Comparisons between groups for continuous data such as body or organ weight by one‐ or two‐way analysis of variance (ANOVA) where appropriate (e.g., diet group alone or diet and sex). Comparison of continuous data that were nested (e.g., analysis on male and female siblings) used restricted maximum likelihood (REML) equations with the dam ID included as a random effect. Appropriateness of any statistical comparison was assessed by visualizing histograms of residuals and further residual (on the y‐axis) plots of (1) fitted values and (2) expected normal quantiles. Any data not normally meeting assumptions for ANOVA or REML (i.e., those with skewed residual errors) were analyzed after log10 transformation. Data are presented as means with standard error of the mean, unless otherwise indicated. 95% confidence intervals may be approximated from the normally distributed data as mean ± ×3 SEM. For most comparisons, we considered *p* < 0.050 as indicating statistical significance, although Bonferroni correction was used when multiple endpoints were being considered (e.g., analysis of FAMEs). Since all measurements were not conducted on all subjects the appropriate experimental *n* for each comparison and statistical method used is indicated in Table and Figure legends. All data were analyzed using Genstat v19 (VSNi, Rothampsted, UK).

## 3 RESULTS

### 3.1 The effects of fructose loading on the liver, kidney and fat deposition in the dam

**FIGURE 1:**
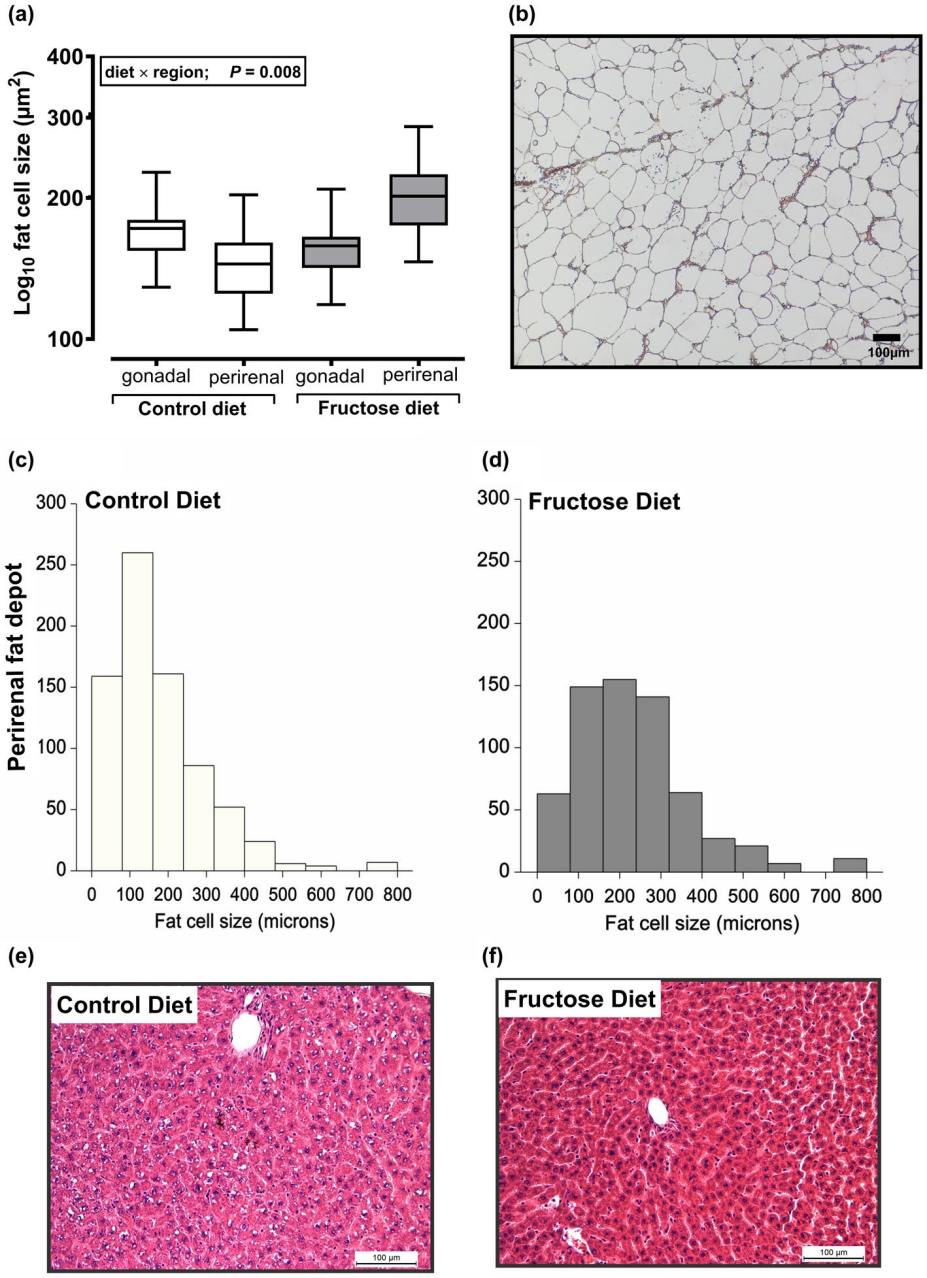
Redistribution of fat in rat dams fed high fructose diet. (a) Fat cell size was measured in three independent sections of adipose tissue using an unbiased randomization technique (3 × 4 box grid, three regions of interest chosen randomly [MS Excel randomize function]). Boxplots represent line at median, boxes are first to third quartiles with whiskers being minimum and maximum values. Data from each individual were summated and analyzed by diet × region; (b) example of a 10 μM section of adipose tissue; (c, d) histograms of fat cell size from control and fructose groups showing no difference in overall distribution of fat cell size and (e, f) H&E‐stained liver sections (5 μm) showing normal hepatocyte morphology with no macro‐ or microvesicular steatosis in periportal, midzonal, or centrilobular regions (i.e., no lipid vacuoles or nuclear displacement), indicating no histological evidence of steatosis on H&E staining. Data are from *n* = 6 dams per group and *n* = 1 offspring per dam. Data analyzed using Genstat v23, Rothampsted, VSNi Ltd. Scale bars: (1b, 1e, and 1f) = 100 μm).

Rat dams responded predictably to fructose loading; supplementing reduced food intake (40.2 ± 1.1 vs. 55.5 ± 1.2 g/day; *p* < 0.001 for fructose vs. control respectively) with increased intake of sweetened beverage (55.8 ± 2.8 vs. 24.6 ± 3.2 mL/day; *p* < 0.001). Taken together, total energy intake tended to be similar prior to conception, but marginally higher through gestation (pooled effect size: before conception, −1.6 ± 0.60; during gestation, +10.3 ± 1.6 kcal/day; *p* < 0.001). Predictably, on day 20 of gestation, fructose‐loaded dams had hepatomegaly with marginally increased retention of tissue water, increased hepatic lipid deposition and marked redistribution of internal fat away from the gonadal depot (i.e., a peripheral site draining to the caudal vena cava) to the perirenal depot (i.e., a central site draining into the hepatic portal vein in rats) (Table 1). Average fat cell size was not different between groups in the gonadal region, but was increased in the perirenal region, suggesting increased lipid storage (Figure 1a).

**TABLE 1:** Effect of fructose‐loading on the pregnant dam's relative organ weight at day 20 gestation.

	Dietary group		*p* Value
	Control	Fructose	
Liver (mg/g)	33.3 ± 1.06	43.2 ± 0.92	<0.001
Liver water content (%)	72.1 ± 0.5	73.0 ± 0.6	0.02
Kidney (mg/g)	4.18 ± 0.13	4.95 ± 0.11	0.001
Perirenal fat (mg/g)	5.44 ± 0.38	8.41 ± 0.32	<0.001
Gonadal fat (mg/g)	19.6 ± 1.89	14.2 ± 1.64	0.008
Hepatic triglyceride (mg/g)	0.96 ± 0.23	2.69 ± 0.93	0.03

*Note*: Dams were euthanized as per standard operating procedures and the wet weight of rats and whole organs immediately measured and expressed relative to dam weight. Data (*n* = 6 dams per dietary group) are mean ± SEM and were analyzed by 1‐way ANOVA (Genstat v18, VSni, Rothampsted, UK).

**FIGURE 2:**
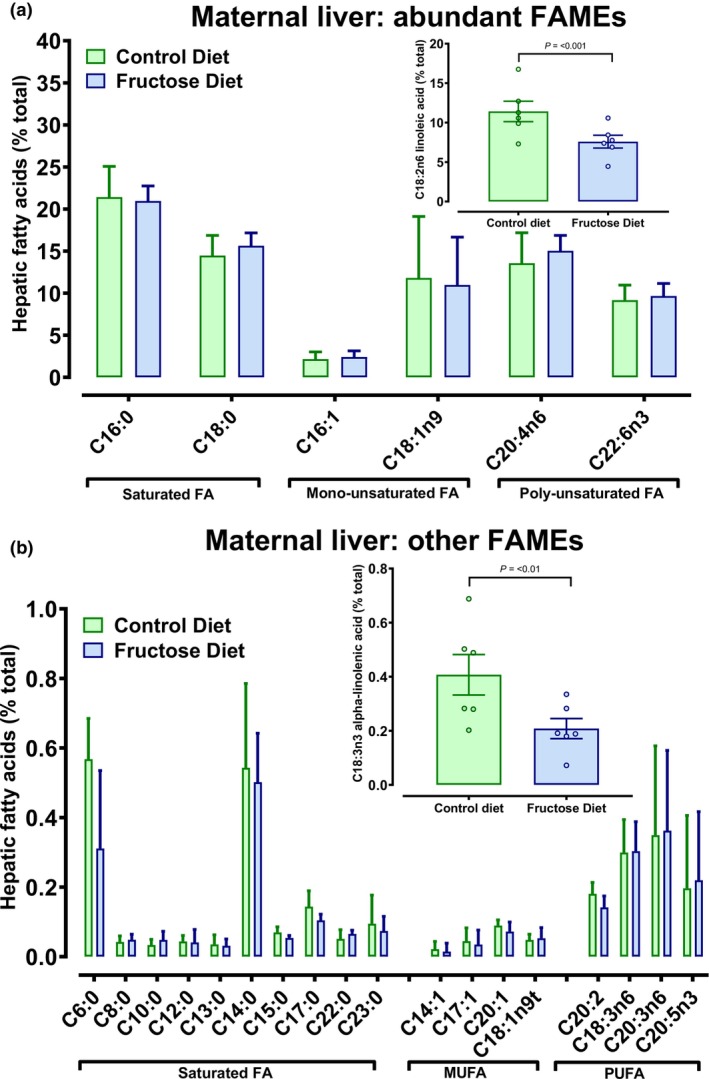
Composition of fatty acid methyl esters (FAME) in livers of rat dams fed high fructose diet. (a) Abundant FAMEs determined by methylation reactions and quantified as individual FA as percentage of total fat extracted (gms %). Linoleic acid (inset) was significantly reduced (*p* < 0.001) in the livers of FD dams; (b) less abundant FAMES with inset, alpha‐linolenic acid being significantly reduced (*p* < 0.01) in the livers of FD dams. FAME were determined in *n* = 6 dams per group. Data analyzed using Genstat v23, Rothampsted, VSNi Ltd).

Further deeper analysis of liver fat composition through derivation of total fatty acid methyl esters (FAMEs) in liver tissue indicated less linoleic (C18:2n6) and alpha‐linolenic (C18:3n3) but no overall difference in hepatic FAMEs (Figure 2). Thus, despite a moderate‐high intake of fructose eliciting clear metabolic effects on the dam, we would consider the overall dietary insult to be relatively mild in comparison to many previously published models of NAFLD, since H&E staining showed no evidence of frank macro−/microvesicular hepatic steatosis nor major differences in the distribution of fat cell size were observed (Figure 1c–f). Increased fluid intake in FD dams elicited major and predictable effects on the kidney to excrete the excess fluid while maintaining plasma osmolality; a notable increase in urine volume with concomitant decreased osmolality was enabled through a marked increase in free water clearance, and to a more limited extent sodium excretion (Table 2). Clearly, increased sweetened water intake elicits major physiological changes to the pregnant dam to cope with the excess of energy and fluid, largely through hepatic and renal adaptation, respectively. Pregnancy is a net anabolic state in which excess nutrient intake and volume expansion are “normal” adaptations to support adequate fetal growth via placental delivery. We therefore considered the effects of fructose‐loading on placental structural anatomy.

**TABLE 2:** Effect of fructose‐sweetened beverage intake on renal function in the d20 pregnant dam.

	Dietary group		*p* Value
	Control	Fructose	
Urine volume (mL·kg BW^−1^)	26.8 ± 4.2	68.8 ± 3.4	<0.001
Plasma osmolality (mosmoles·kg^−1^ H_2_O)	270 ± 3.4	277 ± 7.7	0.43
Urine osmolality (mosmoles·kg^−1^ H_2_O)	1381 ± 18.8	394 ± 58.9	<0.001
Osmolar clearance (mL·min^−1^·kg BW^−1^)	0.10 ± 0.01	0.07 ± 0.01	0.11
Creatinine clearance (mL·min^−1^·kg BW^−1^)	2.12 ± 0.13	2.24 ± 0.37	0.80
Na excretion (μmoles·h^−1^)	32.9 ± 8.6	66.9 ± 20.6	0.20
K excretion (μmoles·h^−1^)	240 ± 56	228 ± 26	0.83
Ca excretion (μmoles·h^−1^)	47.7 ± 14.2	32.0 ± 4.5	0.25
Free water clearance (mL·min^−1^· kg BW^−1^)	26.7 ± 4.2	68.7 ± 3.4	<0.001

*Note*: Renal function was assessed after 24 h in a metabolic crate (see Methods). Elements were measured by ICP‐MS (as mg/L) and mass converted. Data (*n* = 6 dams per dietary group) are mean ± SEM and were analyzed by 1‐way ANOVA (Genstat v18). Statistical significance was accepted at *p* < 0.05.

Abbreviation: BW, body weight.

### 3.2 The effects of fructose loading on the placenta

The precision of the stereologically‐derived estimate for placenta volume was 0.05 (coefficient of error) for all animals (females and males) and was CE, 0.02 for each placental compartment. Fructose loading of the dam had profound negative effects on placenta volume and all compartments including the proportion of vascular tissue (Table 3). This pronounced effect would be expected to have later consequences for all fructose‐exposed offspring and likely underpins the reduction in birthweight observed previously (Gray et al., 2013). Hence, we subsequently characterized similar endpoints in the adult (between 8 and 12 weeks of age) male and female offspring of fructose‐loaded dams.

**TABLE 3:** Effect of fructose‐sweetened beverage intake on placental stereological anatomy.

	Control diet		Fructose diet		Statistics (P‐value)		
	Males	Females	Males	Females	Diet	Sex	D × S
Total volumes (cm^3^)							
Placenta (V_placenta_)	0.38 ± 0.04	0.43 ± 0.04	0.16 ± 0.03	0.12 ± 0.05	<0.001	0.80	0.38
Maternal decidua (V_MD_)	0.035 ± 0.005	0.038 ± 0.005	0.017 ± 0.005	0.012 ± 0.005	0.003	0.94	0.54
Spongio‐trophoblast (V_ST_)	0.06 ± 0.01	0.06 ± 0.01	0.02 ± 0.02	0.01 ± 0.01	<0.001	0.95	0.85
Labyrinth (V_L_)	0.29 ± 0.03	0.33 ± 0.03	0.12 ± 0.03	0.09 ± 0.04	<0.001	0.73	0.33
Labyrinth blood vessels (V_LBV_)	0.104 ± 0.014	0.126 ± 0.014	0.036 ± 0.014	0.029 ± 0.020	<0.001	0.48	0.36
Fractional volumes (%)							
Maternal decidua (V_vMD_)	0.09 ± 0.01	0.08 ± 0.01	0.11 ± 0.02	0.11 ± 0.02	0.39	0.79	0.80
Spongio‐trophoblast (V_vST_)	0.15 ± 0.01	0.15 ± 0.02	0.13 ± 0.02	0.14 ± 0.02	0.38	0.92	0.51
Labyrinth (V_vL_)	0.74 ± 0.02	0.76 ± 0.01	0.75 ± 0.02	0.74 ± 0.02	0.99	0.76	0.33
Labyrinth blood vessels (V_vLBV_)	0.35 ± 0.02	0.37 ± 0.02	0.28 ± 0.02	0.32 ± 0.02	0.004	0.16	0.83

*Note*: Data are mean ± SEM for *n* = 3 males and *n* = 3 females per diet group. Data was analyzed by 2‐way ANOVA with pre‐specified interaction (Genstat v18). Statistical significance was accepted at *p* < 0.05. The volume of each placenta (cm^3^) was estimated by means of the Cavalieri principle (Gundersen et al., 1999; Howard & Reed, 2018; Reed et al., 2010). Fractional volumes (%) of placenta occupied by its respective compartments (maternal decidua, spongio‐trophoblast, labyrinth) and of labyrinth blood vessels were estimated by point counting. Total volumes of each placenta compartment (cm^3^) were estimated by fractional × reference volume (V_placenta_) as described by Gundersen et al. (1999), Howard and Reed (2018), and Mayhew (2008). Two‐way ANOVA indicated a main effect of diet on reducing total and compartment volumes. Fractional vascular volume (V_vLBV_) was also lower in fructose‐exposed placentae.

### 3.3 The effects of fructose loading on the liver, kidney and fat deposition in the offspring

**FIGURE 3:**
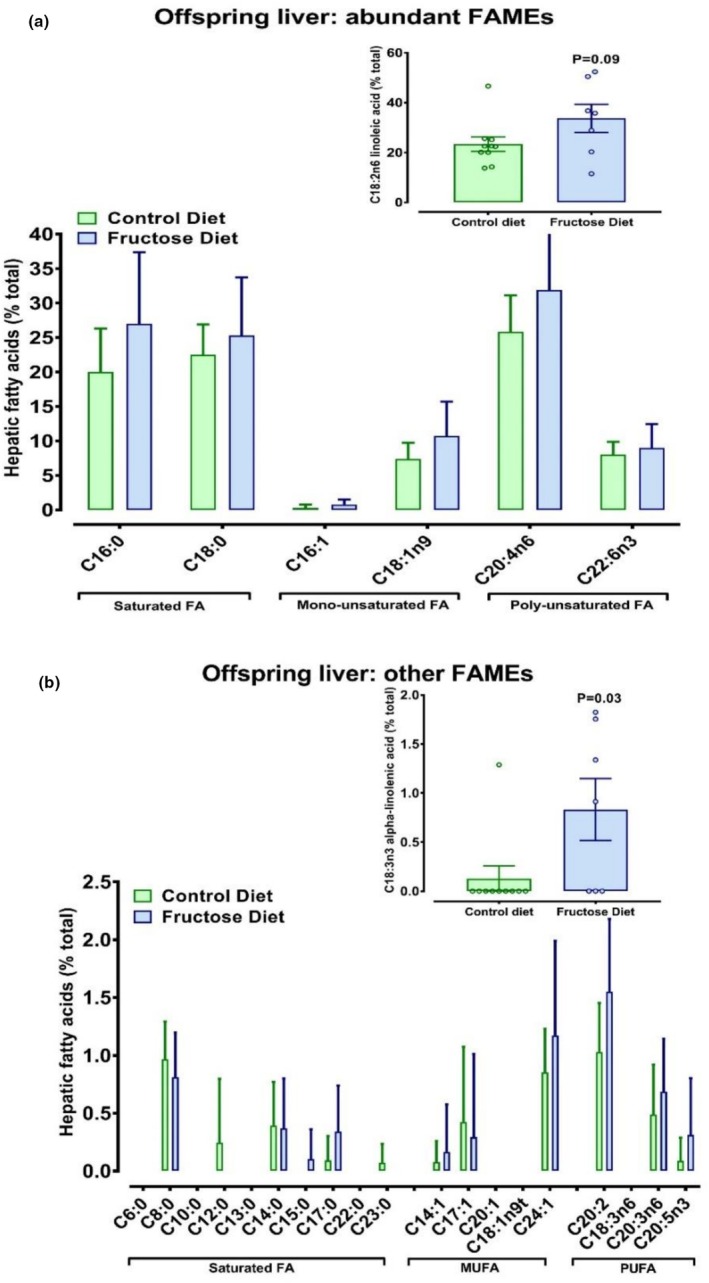
Composition of fatty acid methyl esters (FAME) in livers of offspring from rat dams fed high fructose diet. (a) Abundant FAMEs determined by methylation reactions and quantified as individual FA as percentage of total fat extracted (gms %). Linoleic acid (inset) is shown separately to match Figure 2; (b) less abundant FAMES with inset, alpha‐linolenic acid being significantly increased (*p* = 0.03) in the livers of FD female offspring. FAME were determined in *n* = 6 female offspring per group. Data analyzed using Genstat v23, Rothampsted, VSNi Ltd).

Within each sex there was no overall effect of maternal fructose‐loading on body weight of offspring at 14 weeks of age (CD males, 400 ± 10; FD males, 409 ± 11; CD females, 248 ± 8; FD males, 233 ± 5; P for diet = 0.52). Relative to body weight (mg/kg BW) organ biometry indicated some residual effects of maternal fructose‐loading on adult offspring: both male and female offspring from fructose‐loaded dams tended to retain more tissue water than controls (Table 5). In females, a modest increase in liver water content in fructose‐exposed offspring likely contributed to their greater liver size (Table 4). For the offspring, measurement of total hepatic lipid revealed no residual effects on composition; neither hepatic glycogen nor triglyceride were altered relative to controls (Table 4). Further analysis of hepatic fatty acid methyl esters revealed broad similarity to the profile observed in maternal livers for FAMEs at high concentration (Figure 3a), but a very different profile for those FAMEs at low concentration (Figure 3b). While both linoleic acid (C18:2n6) and α‐linolenic acid (C18:3n3) were decreased in maternal livers of fructose‐loaded rats, they tended to have accumulated in livers of the offspring (Figure 3a,b).

**TABLE 4:** Effect of maternal fructose exposure on offspring biometry.

	Dietary group		*p* Value
	Control diet	Fructose diet	
Males			
Liver (mg/g)	35.8 ± 1.49	35.6 ± 0.92	0.93
Liver water content (%)	69.8 ± 0.3	71.5 ± 0.2	0.03
Kidney (mg/g)	6.77 ± 0.12	6.82 ± 0.12	0.76
Visceral fat (mg/g)	9.34 ± 0.78	9.58 ± 0.78	0.83
Gonadal fat (mg/g)	11.4 ± 0.8	11.4 ± 0.8	0.99
Hepatic triglyceride (mg/g)	0.91 ± 0.11	1.09 ± 0.07	0.20
Hepatic glycogen (mg/g)	63.0 ± 13.3	56.3 ± 7.9	0.67
Females			
Liver (mg/g)	32.7 ± 1.3	37.4 ± 1.4	0.02
Liver water content (%)	70.3 ± 0.3	71.2 ± 0.2	0.02
Kidney (mg/g)	6.84 ± 0.22	7.30 ± 0.24	0.19
Visceral fat (mg/g)	8.09 ± 0.75	7.40 ± 0.82	0.54
Gonadal fat (mg/g)	15.1 ± 1.0	12.4 ± 1.1	0.09
Hepatic triglyceride (mg/g)	1.16 ± 0.16	1.11 ± 0.09	0.98
Hepatic glycogen (mg/g)	57.2 ± 10.7	57.1 ± 6.2	0.98

*Note*: Animals were euthanized as per standard operating procedures and the wet weight of rats and whole organs immediately measured. Data are expressed relative to body weight to normalize differences between males and females. Data are representative of *n* = 5/6 males and females from each dietary group, with one male and one female sibling included from each litter. Values are mean ± SEM and were analyzed by 1‐way ANOVA, blocked by dam to account for shared variance within‐litter (Genstat v18, VSni, Rothampsted, UK).

**TABLE 5:** Effect of maternal fructose‐loading on male and female offspring renal function.

	Dietary group		Statistics
	Control diet	Fructose diet	*p* Value
Male offspring			
Water intake (mL·kg BW^−1^)	69.2 ± 6.4	74.4 ± 6.4	0.58
Urine volume (mL·kg BW^−1^)	25.3 ± 3.4	34.2 ± 3.4	0.03
Plasma osmolality (mosmoles·kg^−1^ H_2_O)	320 ± 10	326 ± 10	0.71
Urine osmolality (mosmoles·kg^−1^ H_2_O)	1981 ± 140	1610 ± 120	0.15
Osmolar clearance (mL·min^−1^·kg BW^−1^)	0.10 ± 0.01	0.11 ± 0.01	0.63
Creatinine clearance (mL·min^−1^·kg BW^−1^)	2.22 ± 1.20	3.75 ± 0.96	0.31
Free water clearance (mL·min^−1^· kg BW^−1^)	24.8 ± 3.2	34.1 ± 2.6	0.06
Female offspring			
Water intake (mL·kg BW^−1^)	103 ± 6.4	121 ± 7.0	0.08
Urine volume (mL·kg BW^−1^)	36.6 ± 3.4	44.9 ± 3.7	0.21
Plasma osmolality (mosmoles·kg^−1^ H_2_O)	319 ± 10	324 ± 11	0.56
Urine osmolality (mosmoles·kg^−1^ H_2_O)	1604 ± 132	1394 ± 126	0.20
Osmolar clearance (mL·min^−1^·kg BW^−1^)	0.11 ± 0.01	0.13 ± 0.01	0.42
Creatinine clearance (mL·min^−1^·kg BW^−1^)	3.11 ± 1.20	3.28 ± 1.06	0.34
Free water clearance (mL·min^−1^· kg BW^−1^)	31.9 ± 2.9	44.6 ± 2.9	0.01^a^

*Note*: Renal function was assessed after 24 h in a metabolic crate (see Methods). Data are representative of *n* = 5/6 males and females per dietary group and are mean ± SEM. All data were analyzed by 1‐way ANOVA, blocked by dam to account for shared variance within‐litter (Genstat v18). Statistical significance was accepted at *p* < 0.05.

Abbreviation: BW, body weight.

^a^ Indicates a significant diet effect within females (Control Diet vs. Fructose Diet) for free water clearance (*p* = 0.01).

## 4 DISCUSSION

### 4.1 Placental structural adaptations to maternal fructose exposure

Design‐based stereology demonstrated a marked reduction in total placental volume in fructose‐exposed dams, with a disproportionate loss of labyrinthine vascular space (lower Vv of blood vessels), indicating vascular rarefaction rather than uniform scaling of compartments. This interpretation aligns with stereology‐based placental morphometry showing that fractional vascular volume is a proxy for exchange capacity (Coan et al., 2004; De Clercq et al., 2020). Within diet‐induced pregnancy models, maternal fructose/high‐sugar exposure has been linked to impaired placental vascularization (Asghar et al., 2016; Thompson & DeBosch, 2021) and altered placental nutrient‐transporter expression/function (Brett et al., 2014; Garcia‐Santillan et al., 2022), consistent with reduced exchange capacity. Our structural findings are consistent with these mechanisms and suggest a plausible upstream constraint on fetal supply. Notably, while later offspring phenotypes exhibit sex‐specific differences, the placental stereology predominantly reflected a diet main effect, suggesting a common antecedent at the interface. We acknowledge that stereology quantifies structure but not perfusion or angiogenic signaling; future studies integrating angiogenic markers and flow surrogates would refine this mechanistic link.

### 4.2 Maternal fructose exposure: Systemic metabolic effects in dams and consequences for offspring

The adverse metabolic consequences of increased consumption of extrinsic sugars, particularly fructose, have been widely reported. Studies show that excessive fructose intake is associated with the development of insulin resistance, dyslipidemia, hypertension, and other cardiovascular disease risk factors (Alam et al., 2022; Hannou et al., 2018; Kanerva et al., 2014; Tappy & Le, 2010).

Most research focuses on the effects of fructose consumption in adult individuals. However, recent studies have explored the impact of maternal fructose intake during pregnancy and lactation, linking it to various metabolic alterations in the mother, with impacts on organs such as the liver, kidneys, and fat accumulation. Castro et al. (2011), for example, observed that Wistar rats fed a diet consisting of 63% fructose for 24 h showed a significant increase in liver fat, highlighting the high lipogenicity of fructose. Castro et al. (2011) reported that Wistar rats fed a 63% fructose diet for 24 h showed a marked rise in hepatic lipid, underscoring fructose's high lipogenicity. By contrast, in our study a moderate–high fructose intake produced clear metabolic effects in dams but constituted a relatively mild dietary insult: H&E‐stained liver sections showed no frank macro‐ or micro‐vesicular steatosis, and adipose histomorphometry revealed no major shift in adipocyte size distribution. These histological observations are concordant with our biochemical data: hepatic triglyceride and glycogen did not differ between groups (Table 4). We acknowledge that H&E is less sensitive than lipid‐specific stains for subtle neutral‐lipid deposition; our conclusion is therefore limited to the absence of steatosis detectable on H&E. Zhao et al. (2025) found an increase in plasma uric acid levels, which may impair renal function and contribute to the development of hypertension. Furthermore, Regnault et al. (2013) demonstrated that excessive fructose intake is associated with increased synthesis of fatty acids and triglycerides, promoting lipogenesis and visceral fat accumulation.

Studies do not focus exclusively on the mother; the impact on offspring health has also been the subject of investigations (Koo et al., 2021; Smith et al., 2022). In 2024, a study conducted by the Federal University of São Paulo (UNIFESP, Brazil) showed that high fructose consumption by parents before conception may increase the risk of cardiometabolic diseases in their offspring. In this study, mice whose parents consumed excessive fructose before mating produced offspring with lower birth weight, elevated triglyceride levels, and insulin resistance. Additionally, these offspring exhibited increased mean blood pressure and impaired baroreceptor sensitivity, a crucial mechanism in blood pressure regulation (dos Santos et al., 2024).

In a similar line of investigation, another study indicated that maternal fructose intake led to alterations in feeding behavior and biochemical parameters in the offspring, suggesting an increased risk of developing metabolic diseases in adulthood (Ramos et al., 2017). A study conducted by the University of Texas Medical Branch (UTMB Health, US) found that pregnant female mice that consumed fructose‐sweetened water produced offspring with a higher risk of developing obesity, hypertension, and metabolic dysfunction in adulthood, particularly in females (Saad et al., 2016). These findings highlight the importance of limiting the intake of fructose‐rich foods and beverages during pregnancy to promote the future health of the offspring.

Importantly, for fructose in particular, these effects in female offspring are exacerbated by further fructose intake, as would naturally tend to occur in human populations (Gray et al., 2016). These differences may relate to hormonal factors and differential expression of genes involved in metabolism and blood pressure regulation between males and females. These findings have significant public health implications, especially considering the increased consumption of processed foods rich in salt and fructose in recent decades.

Studies in animal models have shown that excessive salt intake during pregnancy can negatively impact cardiovascular health in the offspring (Ge et al., 2021; Kagota et al., 2017; Tain & Hsu, 2022). Although the interaction between maternal salt and fructose consumption has not been widely studied, it is plausible that the combination of these factors could exacerbate adverse effects in the offspring, considering shared pathophysiological mechanisms such as oxidative stress and systemic inflammation.

## 5 CONCLUSIONS

This study has demonstrated that maternal intake of fructose during gestation produced structural changes in the placenta—including reduced total volume and vascular (exchange) fraction. These findings are consistent with fructose‐driven developmental programming and highlight the need for preconception and antenatal dietary guidance to minimize placental maladaptation and reduce the risk of developing non‐communicable diseases in future generations.

## AUTHOR CONTRIBUTIONS

D.S.G. and C.G. designed research; C.G., A.A.C. T.S.S., and D.S.G. conducted the research; D.S.G., C.G., T.S.S., and A.A.C. wrote the paper; A.L.L. and F.V.L.L. critically evaluated the content. A.A.C. and D.S.G. have primary responsibility for its final content.

## FUNDING INFORMATION

This work was supported by a BBSRC doctoral training grant to C.G. (50:50 with The University of Nottingham). Grant support from The Nutricia Research Foundation and The School of Veterinary Medicine and Science is gratefully acknowledged.

## CONFLICT OF INTEREST STATEMENT

None declared.

## Data Availability

The datasets generated during the current study are available from either corresponding author on reasonable request.
